# Androgen Receptor Upregulates Mucosa-Associated Lymphoid Tissue 1 to Induce NF-κB Activity via Androgen-Dependent and -Independent Pathways in Prostate Carcinoma Cells

**DOI:** 10.3390/ijms24076245

**Published:** 2023-03-26

**Authors:** Kang-Shuo Chang, Syue-Ting Chen, Hsin-Ching Sung, Shu-Yuan Hsu, Wei-Yin Lin, Chen-Pang Hou, Yu-Hsiang Lin, Tsui-Hsia Feng, Ke-Hung Tsui, Horng-Heng Juang

**Affiliations:** 1 Department of Anatomy, College of Medicine, Chang Gung University, Kwei-Shan, Tao-Yuan 33302, Taiwan; 2 Graduate Institute of Biomedical Sciences, College of Medicine, Chang Gung University, Kwei-Shan, Tao-Yuan 33302, Taiwan; 3 Department of Internal Medicine, Chang Gung Memorial Hospital-Linkou, Kwei-Shan, Tao-Yuan 33302, Taiwan; 4 Department of Urology, Chang Gung Memorial Hospital-Linkou, Kwei-Shan, Tao-Yuan 33302, Taiwan; 5 School of Nursing, College of Medicine, Chang Gung University, Kwei-Shan, Tao-Yuan 33302, Taiwan; 6 Department of Urology, Shuang Ho Hospital, New Taipei City 235041, Taiwan; 7 Department of Medicine, College of Medicine, Taipei Cancer Center, Taipei Medical University, Taipei 11031, Taiwan

**Keywords:** prostate, androgen receptor, ARv7, MALT1, PSA, NDRG1

## Abstract

The androgen-dependent or -independent pathways are regarded as primary therapeutic targets for the neoplasm of the prostate. Mucosa-associated lymphoid tissue 1 (MALT1) acting as a paracaspase in the regulation of nuclear factor κB (NF-κB) signal transduction plays a central role in inflammation and oncogenesis in cancers. This study confirmed the potential linkages between androgen and NF-κB activation by inducing MALT1 in the androgen receptor-full length (ARFL)-positive LNCaP and 22Rv1 prostate cancer cells. Although androgen did not stimulate MALT1 expression in AR-null or ectopic ARFL-overexpressed PC-3 cells, the ectopic overexpression of the AR splicing variant 7 (ARv7) upregulated MALT1 to activate NF-κB activities in 22Rv1 and PC-3 cells. Since the nuclear translocation of p50 and p65 was facilitated by ARv7 to motivate NF-κB activity, the expressions of MALT1, prostate-specific antigen (PSA), and N-myc downstream regulated 1 (NDRG1) were therefore induced in ectopic ARv7-overexpressed prostate cancer cells. Ectopic ARv7 overexpression not only enhanced 22Rv1 or PC-3 cell growth and invasion in vitro but also the tumor growth of PC-3 cells in vivo. These results indicate that an androgen receptor induces MALT1 expression androgen-dependently and -independently in ARFL- or ARv7-overexpressed prostate cancer cells, suggesting a novel ARv7/MALT1/NF-κB-signaling pathway may exist in the cells of prostate cancer.

## 1. Introduction

A previous study has concluded that reducing androgen signaling determines the progression of prostate cancer [1]. However, the emerging castration-resistant prostate cancer (CRPC) during cell-autonomous androgen receptor (AR) signaling complicates the progression of prostate cancer. The modulation of AR signaling is partly through the amplification, mutation, splice variant of the AR, as well as by its coactivator or corepressor. Additionally, certain bypass-signaling pathways, such as NF-κB, growth factor, and cytokines, in the prostate cells involved in the enhancement of AR signaling have been underscored [2]. The full-length AR (ARFL) protein shares similar structures with other steroid hormone nuclear receptors which have four functional domains including the N-terminal activation domain, the central DNA-binding domain, the hinge domain, and the C-terminal ligand-binding domain [3,4]. However, a prior study has found multiple splicing variant ARs (AR-Vs) in prostate cancer cells, xenografts, and tumors [5]. A total of 22 AR variants have been illustrated and validated as having constitutively active AR-Vs, conditionally active AR-Vs, and inactive AR-Vs [6,7]. The AR variant 7 (ARv7), which lacks the conserved C-terminal ligand binding domain, was detected in various tumors and circulating tumor cells of CRPC patients [8,9]. Moreover, studies using the cistrome and transcriptome analyses in CRPC revealed that both ARFL and ARv7 have their own unique transcriptional activities [10,11].

NF-κB acts as a link between inflammation and CRPC progression [12,13]. Even though the association of androgen and NF-κB signaling has been well discussed, the sustained activation of NF-κB has been reported in androgen-independent prostate cancer cells [14,15,16,17]. Studies have illustrated the crosstalk between AR and NF-κB in which the transcriptome in the cells of prostate cancer may be reprogrammed [18,19]. However, a recent report showed a contrary relevance between AR and NF-κB by comparing human prostate cancer cells and mouse prostate tissues [20]. Interestingly, one study concluded that the elevation of ARv7 expression, not ARFL expression, may be involved in the upregulation of NF-κB signaling in the late versus the early stage of benign prostatic hyperplasia [21].

Similar to caspases, mucosa-associated lymphoid tissue protein 1 (MALT1), a paracaspase with arginine-specific proteolytic activity, enhances NF-κB activation by degrading the NF-κB negative regulator [22,23]. Most studies focus on the paracaspase activity of MALT1 and NF-κB signaling in the immunity of lymphoma. The therapeutic targeting of MALT1 protease activity is a potential approach for the treatment of lymphomas or an effective strategy for treating neoplastic and inflammatory disorders associated with dysregulated NF-κB signaling [24,25]. However, the oncogenic role of MALT1 in certain non-lymphoid solid tumors, including prostate neoplasm, has been demonstrated [26,27,28,29,30].

Prostate-specific antigen (PSA) is a well-known tumor marker of prostate cancer [31]. The androgen directly regulates the expression of PSA by binding to the AR and facilitates AR binding to the androgen response element (ARE) within the enhancer region of the PSA gene [32]. However, previous studies have found that NF-κB activates PSA expression in androgen-dependent and -independent prostate cancer cells [33,34]. A recent study has elucidated that caffeic acid phenethyl ester, a bioactive component of propolis with antagonistic activity to androgen and NF-κB, blocked MALT1 expression via androgen and NF-κB signaling, suggesting that MALT1 may have a critical role in the crosstalk between AR and NF-κB signaling in human prostate cancer cells in vitro and in vivo [35]. However, the molecular mechanisms of the regulation of AR on MALT1 in androgen-dependent and -independent prostate cancer cells have yet to be clearly established.

The aims of this study are to unveil whether the effect of full-length androgen receptor (ARFL) and splicing variant 7 AR (ARv7) on the expression of MALT1 is androgen-dependent or androgen-independent, and, furthermore, to evaluate the potential ARv7/MALT1/NF-κB-signaling pathway in human prostate cancer cells.

## 2. Results

### 2.1. Androgen Induces MALT1 Expression in LNCaP and 22Rv1 Prostate Cancer Cells

**Figure 1:**
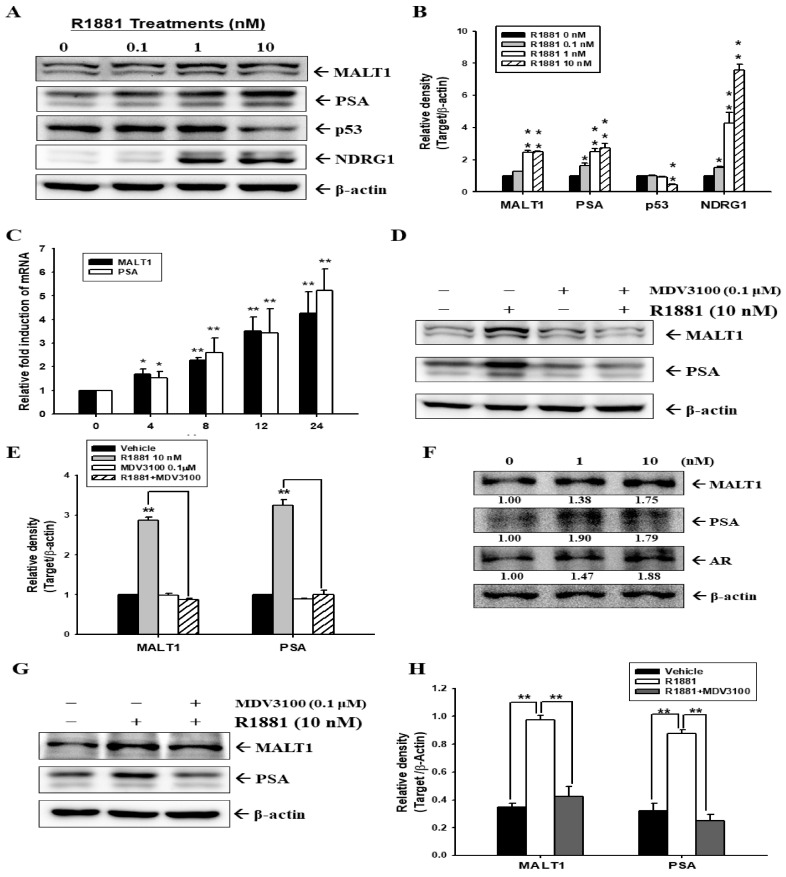
Effect of androgen on MALT1 expression in AR-positive prostate carcer cells. The LNCaP cells were treated with various concentrations of R1881 for 24 h. The cells were lysed and then MALT1, PSA, p53, NDRG1, and β-actin were determined by immunoblot assays (**A**) and the quantitative data were expressed as the intensity of protein bands of the target proteins/β-actin relative to the control vehicle-treated group (**B**). (**C**) The LNCaP cells were treated with 10 nM of R1881 as indicated. The mRNA levels of MALT1 and PSA relative to time 0 were determined by RT-qPCR assays. The LNCaP were cotreated with/without 10 nM R1881 and/or 0.1 μM MDV3100 as indicated for 24 h. The cells were lysed, then MALT1, PSA, and β-actin were determined by immunoblotting (**D**) and quantitative analysis (**E**). (**F**) The 22Rv1 cells were treated with various concentrations of R1881 for 24 h. The cells were lysed and then MALT1, PSA, AR, and β-actin were determined by immunoblotting. The numbers indicate the ratio of target proteins/β-actin in relation to vehicle treatment. The 22Rv1 cells were cotreated with/without 10 nM R1881 and/or 0.1 μM MDV3100 as indicated for 24 h. The cells were lysed, then MALT1, PSA, and β-actin were determined by immunoblotting (**G**) and a quantitative analysis was presented as a relative density of MALT or PSA/β-actin (**H**). * *p* < 0.05, ** *p* < 0.01.

To determine the role of the androgen analog, R1881, on the expression of MALT1 in AR-positive prostate cancer cells, we treated two ARFL-positive prostate cancer cells, LNCaP and 22Rv1, with various concentrations of R1881 for 24 h. The androgen analog (R1881) treatment dose-dependently enhanced MALT1, PSA, and NDRG1 expression but blocked p53 expression in LNCaP cells (Figure 1A). A quantitative analysis is presented in Figure 1B. The RT-qPCR assays indicated further that R1881 treatment induced MALT1 and PSA gene expression at the transcriptional level in LNCaP cells in a time-dependent manner (Figure 1C). The results of immunoblot assays (Figure 1D) and the quantitative analysis (Figure 1E) displayed that R1881-induced upregulations of MALT1 and PSA protein levels were attenuated by cotreatment with MDV3100, an AR antagonist, indicating that the androgen-induced gene expression of PSA and MALT1 was dependent on the AR at the transcriptional level. The immunoblot assays also exhibited that R1881 treatment enhanced MALT1, PSA, and AR expression in androgen-independent 22Rv1 cells at the transcriptional level (Figure 1F,G). The quantitative analysis of the immunoblot assays are displayed in Figure 1H. Taken together, these results indicated that androgen upregulates both MALT1 and PSA expression in either ARFL-positive androgen-dependent LNCaP cells or androgen-independent 22Rv1 cells.

### 2.2. Androgen Upregulates MALT1 to Promote PSA Expression in LNCaP Cells

**Figure 2:**
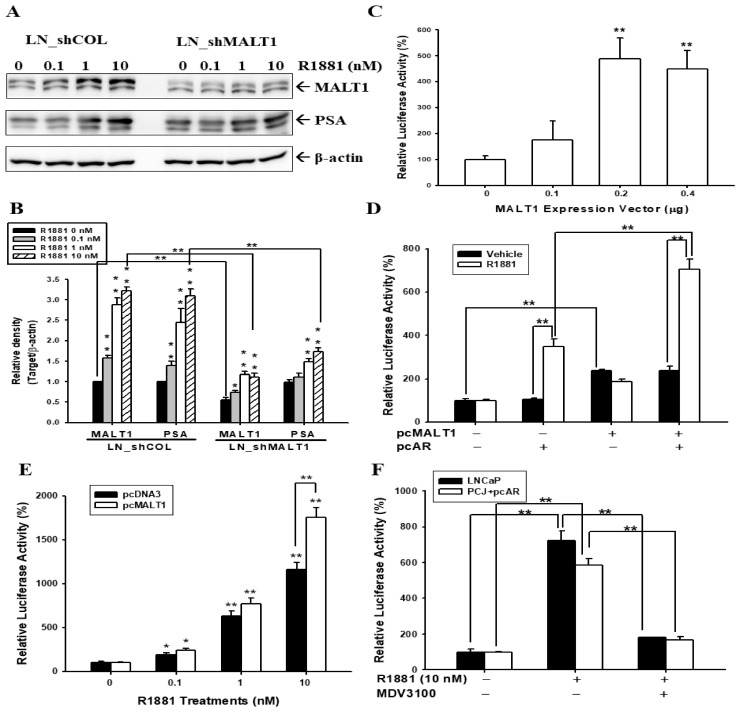
Effects of androgen and MALT1 on PSA expression in androgen-dependent LNCaP prostate cancer cells. The mock-knockdowned LNCaP (LN_shCOL) and MALT1-knockdowned LNCaP (LN_shMALT1) cells were treated with various concentrations of R1881 as indicated for 24 h. The cells were lysed, then MALT1, PSA, and β-actin were determined by (**A**) immunoblot assays and (**B**) quantitative analysis. (**C**) The reporter activity of the PSA reporter vector was cotransfected with various concentrations of MALT1 expression vectors in LNCaP cells. (**D**) The reporter activity of the PSA reporter vector was cotransfected with MALT1 and/or AR expression vectors and treated with/without 10 nM R1881 in PCJ cells. (**E**) The reporter activity of the PSA reporter vector was cotransfected with MALT1 expression vectors (white bars) or control vectors (pcDNA3, black bars), and then treated with various concentrations of R1881 as indicated for 24 h in the LNCaP cells. The data are presented as mean percentages (±SE, n = 6) relative to the control vehicle-treated group. (**F**) The luciferase activity of the PSA reporter vector was treated with/without 10 nM R1881 and 0.1 μM MDV3100 as indicated for 24 h in LNCaP or transient overexpressed AR PCJ cells. * *p* < 0.05, ** *p* < 0.01.

To determine whether androgen enhanced PSA expression via the upregulation of MALT1, we applied androgen to the mock-transduced LNCaP (LN_shCOL) cells and also the MALT1-knockdowned LNCaP (LN_shMALT1) cells. Interestingly, the results of the immunoblot assays (Figure 2A) and the quantitative analysis (Figure 2B) confirmed that MALT1 protein levels in the LN_shMALT1 cells were knockdowned by 40% as compared to the LN_shCOL cells. Moreover, the results also revealed that R1881 upregulated MALT1 and PSA expression in the LN_shCOL cells; whereas the knockdown of MALT1 in the LN_shMALT1 cells attenuated the R1881 effect on PSA expression. The results of reporter assays indicated that transient cotransfection with various concentrations of MALT1 expression vectors induced PSA promoter activity dose-dependently in the LNCaP cells (Figure 2C). Further reporter assays using the subculture cell line of PC-3 (PCJ) cells indicated that MALT1 induced PSA luciferase activity without 10 nM R1881 treatment; however, R1881 only upregulated PSA luciferase activity when the cells were cotransfected with an ARFL expression vector. MALT1 cotransfection also enhanced the R1881-induced PSA luciferase activity in ARFL-transfected PCJ cells (Figure 2D). The reporter assays indicated that R1881 induced PSA luciferase activity dose-dependently in LNCaP cells; moreover, the cotransfection of the MALT1 expression vector enhanced R1881-induced PSA luciferase activity in LNCaP cells (Figure 2E). Meanwhile, the MDV3100 treatment blocked the effect of R1881 activation on the luciferase activity of the PSA reporter vector in LNCaP and ARFL-transfected PCJ cells (Figure 2F).

### 2.3. Androgen Upregulates MALT1 to Promote NF-κB Activation in LNCaP Cells

**Figure 3:**
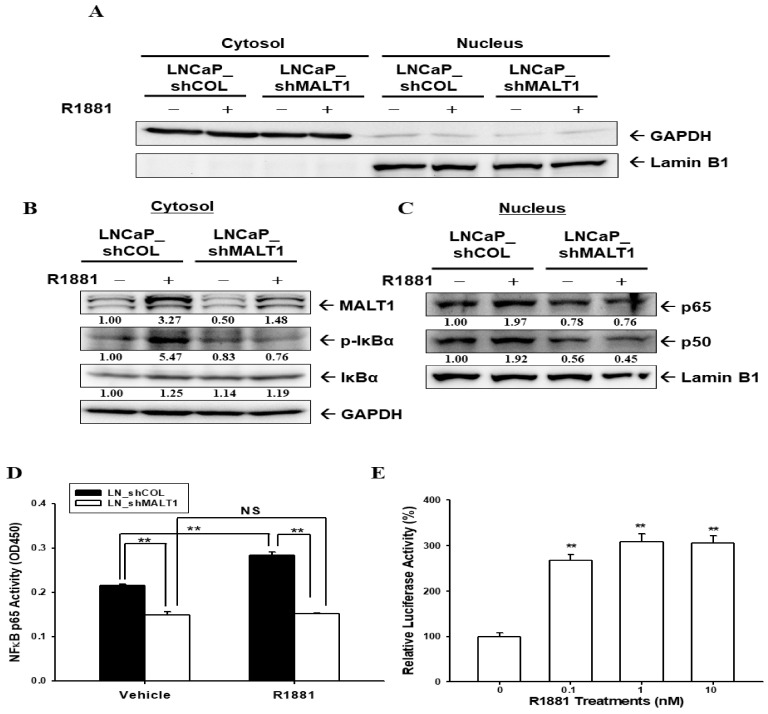
Effect of androgen on NF-κB signaling is dependent on MALT1 in androgen-dependent LNCaP prostate cancer cells. The LN_shCOL and LN_shMALT1 cells were treated with or without 10 nM of R1881 for 24 h. (**A**) The expression of GAPDH in cytosol and Lamin B1 in the nucleus were determined by immunoblotting after the separation of nuclear and cytoplasmic fractions as indicated. The expression of MALT1, IκBα, phospho-IκBα, and GAPDH in the cytosol fraction (**B**), as well as p65, p50, and Lamin B1 in the nuclear fraction (**C**), were determined by immunoblotting. The numbers indicate the ratio of target proteins/GAPDH or Lamin B1 in relation to vehicle treatment. (**D**) The NF-κB (p65)-binding activity in the LN_shCOL and LN_shMALT1 cells after treatment with R1881 for 18 h. (**E**) The reporter activity of the MALT1 reporter vector was treated with various concentrations of R1881 as indicated for 24 h in the 22Rv1 cells. The data are presented as mean percentages (±SE, n = 6) relative to the control vehicle-treated group. ** *p* < 0.01, NS represents no significance.

Next, we investigated whether androgen-induced MALT1 expression facilitates the nuclear translocation of NF-κB signaling in AR-positive prostate cancer cells. The immunoblot assays confirmed that the cytoplasmic and nuclear fractions from R1881-treated AR-positive LNCaP cells (LN_shCOL and LN_shMALT1) were separated (Figure 3A). R1881 treatment induced MALT1 expression and IκBα phosphorylation in cytoplasmic fractions (Figure 3B), thereby eliciting both p65 and p50 protein levels in the nucleus (Figure 3C). These effects were attenuated in MALT1-knockdowned LNCaP cells. Similar results showed in the LNCaP cells that the knockdown of MALT1 blocked NF-κB activity (Figure 3D). The results of reporter assays indicated that R1881 enhanced the luciferase activity of the MALT1 reporter vector in 22Rv1 cells (Figure 3E). Overall, these results demonstrated that R1881 activates NF-κB signaling via MALT1 in prostate cancer cells.

### 2.4. Androgen Does Not Induce MALT1 Expression in Ectopic ARFL-Overexpressed PC-3 Cells

**Figure 4:**
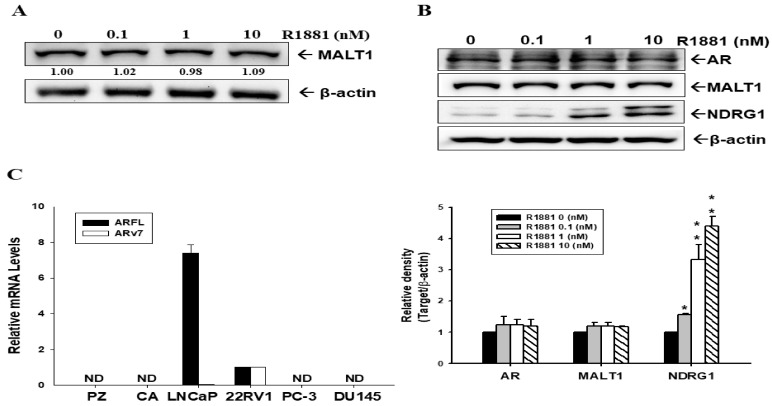
Androgen does not affect MALT1 expression in PC-3 cells and ectopic ARFL-overexpressed PC-3 cells. (**A**) The PC-DNA cells were treated with various concentrations of R1881 as indicated for 24 h. The cells were lysed, and the MALT1 and β-actin expression was determined using immunoblot assays. The numbers indicate the ratio of target proteins/β-actin in relation to vehicle-treatment. (**B**) The PC-ARFL cells were treated with various concentrations of R1881 as indicated for 24 h. The cells were lysed and analyzed for AR, MALT1, NDRG1, and β-actin using immunoblot assays (top), as above. The quantitative data were expressed as the intensity of protein bands of the target proteins/β-actin relative to the control vehicle-treated group (bottom). (**C**) The mRNA levels of ARFL and ARv7 in the prostate cell lines, as indicated, were determined by RT-qPCR analysis (PZ: PZ-HPV-7, CA: CA-HPV-10). The mRNA levels of ARFL and ARv7 in 22Rv1 cells are regarded as 1, and ND represents non-detectable. * *p* < 0.05, ** *p* < 0.01.

However, immunoblot assays revealed that MALT1 expression was not influenced by androgen treatment in AR-null PC-3 cells (Figure 4A). Although androgen did indeed induce NDRG1 expression dose-dependently in ectopic ARFL-overexpressed PC-3 (PC-ARFL) cells, indicating that the expression of MALT1 was not affected by androgen in PC-ARFL cells, even preserving the function of ARFL (Figure 4B). Since the results in Figure 4B confirmed that R1881 did not affect the MALT1 expression in PC-ARFL cells, we continued to evaluate whether the splicing variant 7 AR (ARv7) was involved in the modulation of MALT1 under androgen-free conditions. Firstly, we determined the mRNA levels of ARFL and ARv7 in prostate cell lines. The results of the RT-qPCR analysis showed that the mRNA levels of ARFL and ARv7 were only expressed in LNCaP and 22Rv1 cells (Figure 4C).

### 2.5. Ectopic ARv7 Overexpression Enhances MALT1 to Promote NF-κB Activation in PC-3 and 22Rv1 Cells

**Figure 5:**
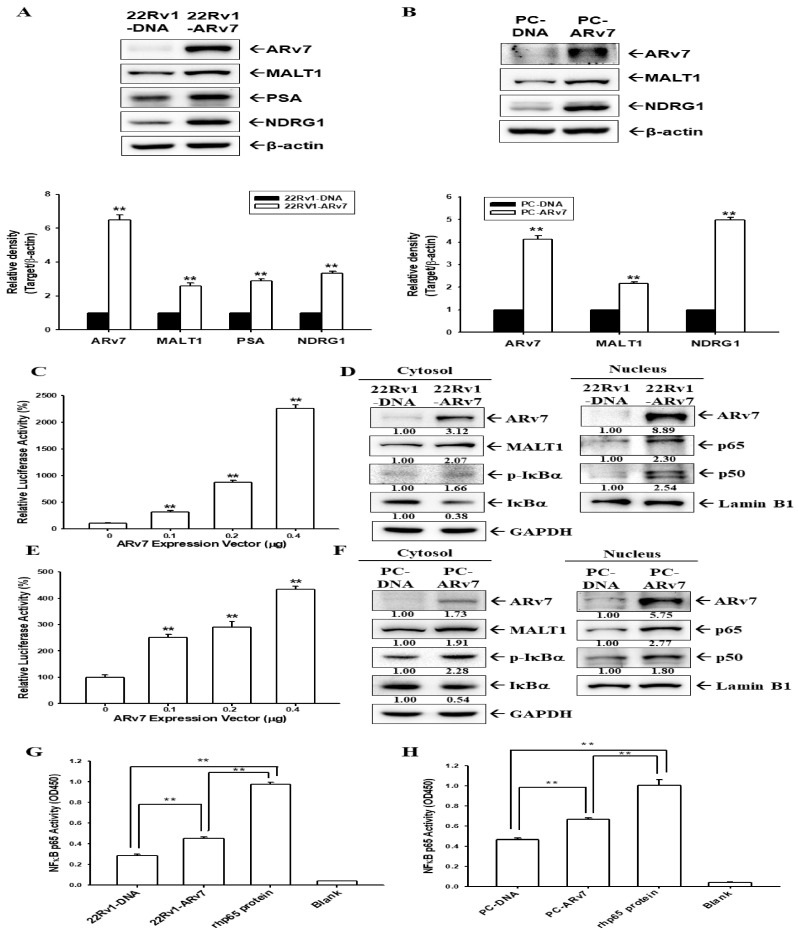
Ectopic overexpression of ARv7 upregulates MALT1 expression to enhance NF-κB activation in PC-3 and 22Rv1 cells. An immunoblot assay of the expression of ARv7, MALT1, PSA, NDRG1, and β-actin in 22Rv1 (**A**) and PC-3 (**B**) cells. The quantitative data of the immunoblot assays were presented as the mean fold induction (±SE, n = 3) of the target proteins/β-actin relative to the mock-transfected group. The reporter activity of the MALT1 reporter vector was cotransfected with various concentrations of the ARv7 expression vectors, as indicated, for 24 h in the 22Rv1 (**C**) and PC-3 (**E**) cells. The data are presented as mean percentages (±SE, n = 6) relative to the control vehicle-treated group. The expression of ARv7, MALT1, IκBα, phospho-IκBα, p65, p50, Lamin B1, and GAPDH in the 22Rv1 (**D**) and PC-3 (**F**) cells after the separation of the nuclear and cytoplasmic fractions, as indicated, were determined by immunoblot assays. The numbers indicate the ratio of target proteins/GAPDH or Lamin B1 in relation to the mock-transfected group. The NF-κB (p65)-binding activity in the 22Rv1 (**G**) and PC-3 (**H**) cells after stably transfecting with ARv7. ** *p* < 0.01.

The ectopic overexpressed ARv7 in the androgen-independent prostate cancer cells, 22Rv1 and PC-3, were cloned stably. Immunoblot assays and a quantitative analysis revealed that the ectopic overexpression of ARv7 upregulated the expression of MALT1, NDRG1, or PSA in 22Rv1 (Figure 5A) and PC-3 (Figure 5B) cells. The induction of ARv7 in MALT1 expression occurs at transcriptional levels, since reporter assays found that cotransfection with the ARv7 expression vector enhanced the reporter activity of the human MALT1 reporter vector in 22Rv1 (Figure 5C) and PC-3 (Figure 5E) cells. Furthermore, the effect of ARv7 on the modulation of NF-κB signaling in 22Rv1 and PC3 cells was investigated. The nuclear and cytoplasmic fractions were separated and determined by Lamin B1 and GAPDH, respectively, showing that the ectopic overexpression of ARv7 enhanced cytosolic MALT1 protein, with the upregulation of p-IκBα protein expression in the cytoplasmic fraction and NF-κB (p50, p65) in the nuclear fraction, respectively (Figure 5D,F). Moreover, the NF-κB activity was upregulated in 22Rv1-ARv7 (Figure 5G) and PC-ARv7 (Figure 5H) cells compared with mock-overexpressed 22Rv1 (22Rv1-DNA) and PC-3 (PC-DNA) cells determined by the NF-κB (p65) transcription factor binding assays.

### 2.6. Ectopic ARv7 Overexpression Promotes Cell Proliferation and Invasion in PC-3 and 22Rv1 Cells

**Figure 6:**
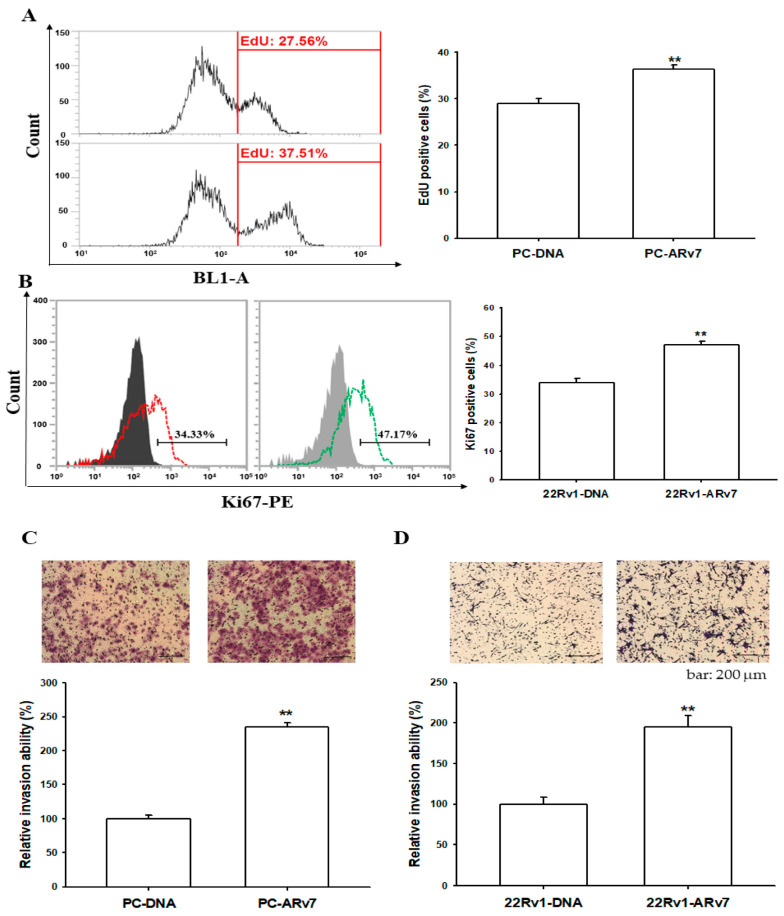
Effects of ectopic-overexpressed ARv7 on cell proliferation and invasion in PC-3 and 22Rv1 cells. (**A**) The proliferation ability of PC-DNA and PC-ARv7 cells was determined through flow cytometry using the Click-iT EdU flow cytometry kit (±SE, n = 4). (**B**) The proliferation ability of 22Rv1-DNA and 22Rv1-ARv7 cells was determined through flow cytometry using the Ki67 flow cytometry kit (±SE, n = 4). The invasion ability of PC-3 (**C**) and 22Rv1 (**D**) cells after stable transfection with the mock vector or ARv7 expression vector was determined through in vitro Matrigel invasion assays (±SE, n = 3). The bar line represents 200 μm. ** *p* < 0.01.

To further evaluate the role of the ectopic overexpression of ARv7 in the growth of prostate cancer cells, we used the EdU proliferation assay to determine the growth of PC-DNA and PC-ARv7 cells and the Ki67 proliferation assay to assess the growth of 22Rv1-DNA and 22Rv1-ARv7 cells. The cell proliferation assays revealed that ARv7-overexpressed PC-3 (PC-ARv7) and 22Rv1 (22Rv1-ARv7) cells possessed much higher proliferative rates compared to mock-transfected PC-3 (PC-DNA) and 22Rv1 (22Rv1-DNA) cells, respectively (Figure 6A,B). In addition, the Matrigel invasion assays showed that the overexpression of ARv7 significantly enhanced cell invasion in both the PC-3 (Figure 6C) and 22Rv1 (Figure 6D) cells.

### 2.7. Ectopic ARv7 Overexpression Enhances Tumor Growth of PC-3 Cells in Xenograft Animal Study

**Figure 7:**
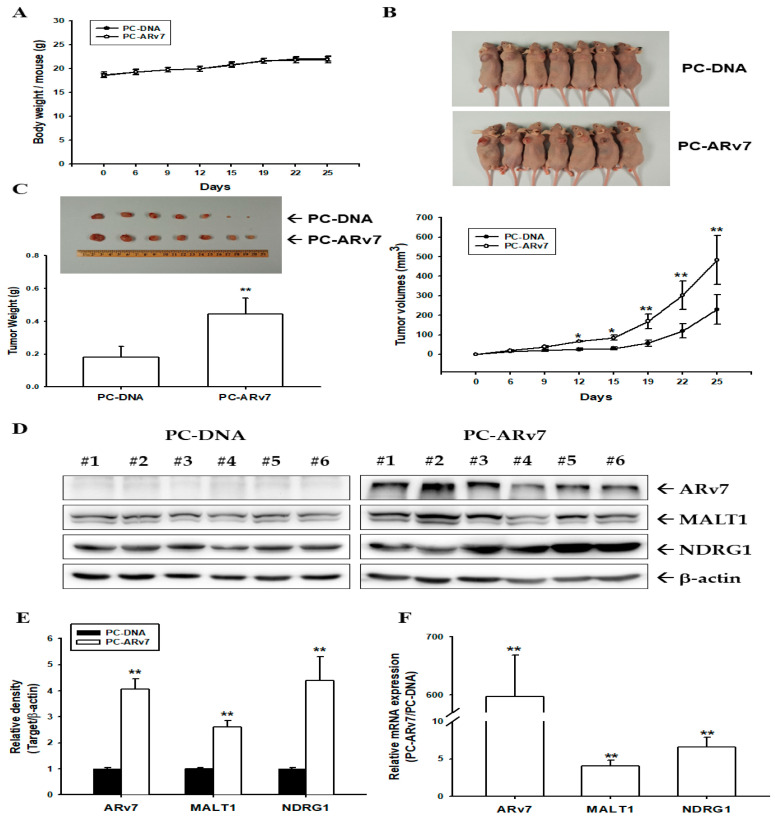
Ectopic overexpression of ARv7 enhances tumorigenesis of PC-3 cells in xenograft mice models. The athymic male nude mice were subcutaneously injected with mock-overexpressed (PC-DNA) or ectopic-overexpressed ARv7 (PC-ARv7) cells for 25 days. (**A**) The average body weight (mean ± SE) of the mice during the experimental period. Photograph of the representative xenografted mice and tumors (**B**, **top**) and tumor sizes (**B**, **bottom**), which were measured every 3 days. The mice were sacrificed on the 25th day and the tumors were excised. (**C**) The quantitative data (mean ± SE) of the tumor weight of the PC-DNA and PC-ARv7 groups. (**D**) Whole-cell lysates of tumor samples from the PC-DNA and PC-ARv7 groups were subjected to immunoblot assays for ARv7, MALT1, NDRG1, and β-actin. (**E**) The quantitative data of the immunoblot assays are presented as the mean fold induction of ARv7, MALT1, and NDRG1 relative to the PC-DNA group. (**F**) The mRNA levels of ARv7, MALT1, and NDRG1 in the xenografted tumors were analyzed by RT-qPCR assays (±SE, n = 3). * *p* < 0.05, ** *p* < 0.01.

To evaluate the growth effect of the ectopic overexpression of ARv7 on ARv7-null PC-3 prostate cancer cells in vivo, PC-DNA and PC-ARv7 cells were xenografted subcutaneously in athymic nude mice, respectively. The body weight of both groups did not significantly differ during the experimental period (Figure 7A). The solid tumors derived from the PC-DNA and PC-ARv7 groups were estimated, and the tumor size in both groups was determined every 3 days (Figure 7B, top). After 25 days of xenograft, the tumor sizes (Figure 7B, bottom) were 52% lower (230.21 ± 75.18 vs. 483.84 ± 124.67 mm^3^) and the tumor weights (Figure 7C) were 59% lower (0.1823 ± 0.0640 vs. 0.4432 ± 0.0992 g) in the PC-DNA group than those in the PC-ARv7 group, respectively. Moreover, in the PC-ARv7-xenografted groups the levels of ARv7, MALT1, and NDRG1 proteins, as well as the mRNA levels, increased compared to the mock-transfected PC-3 groups, as determined by immunoblot (Figure 7D,E) and RT-qPCR (Figure 7F) assays. Taken together, the findings indicate that the ectopic overexpression of ARv7 enhanced PC-3 cell growth both in vitro and in vivo.

## 3. Discussion

The role of androgen in the development of the human prostate has been well established; meanwhile, for the past decades androgen-deprivation therapy has become the standard care for metastatic prostate cancer [36]. However, modern clinical practice has found that recurrent castration-resistant prostate cancer may relate to other alternative pathways, whether in an androgen-dependent or androgen-independent manner [2]. NF-κB signaling is a well-known signature of the progression of prostate cancer [37]. Reports concerning NF-κB signaling in prostate cancer, either androgen-dependently or androgen-independently, still remain inconclusive. Early studies have indicated that androgen-independent prostate cancer cells and xenografts present an elevated constitutive NF-κB activity, suggesting a negative regulation between AR and NF-κB signaling [34,38]. In addition, a recent study showed that the AR and the NF-κB signal are antagonistic to each other in vitro and in silico [20]. However, several studies have exhibited a positive crosstalk between AR and NF-κB signaling in prostate cancer [13,17,18,19].

The results from this study indicate that androgen-stimulated MALT1 activates NF-κB signaling in the ARFL-positive prostate cancer cell lines, LNCaP and 22Rv1 (Figure 1). These results may support one previous study which suggested highly expressed MALT1 in both LNCaP and 22Rv1 compared to the other ARFL-null prostate cancer cell lines, PC-3 and DU145 [30]. However, a prior study using a gene profile analysis with a PCR methylation array panel revealed that androgen induced the hypermethylation of DNA in the MALT1 promoter of LNCaP cells [39]. The opposite effect of androgen on p53 and MALT1 in LNCaP cells presented in Figure 1A is in agreement with one previous study on AR/p53/NF-κB signaling in human prostate cancer cells [35]. This study illustrated further that androgen induces MALT1 at the transcriptional level, and the ligand-binding domain-targeted antiandrogen, MDV3100, inhibits the effect of androgen. Immunoblot assays indicated that MALT1 knockdowned in LNCaP cells downregulated 40–50% of MALT1 expression as compared to mock-knockdown LNCaP cells (Figure 2 and Figure 3), which is similar to our previous study on PC-3 cells [30]. Furthermore, the knockdown of MALT1 suppressed the effect of R1881 on PSA expression in LNCaP cells. A transient transfected MALT1 expression vector induced PSA reporter activity and androgen syngeneic-upregulated PSA reporter activity in cotransfected ARFL and MALT1 in PCJ, a subculture of PC-3 cells, implying that MALT1 upregulated PSA expression in an androgen/ARFL and androgen-independent manner (Figure 2).

The activation of NF-κB inducing PSA expression in androgen-dependent and androgen-independent prostate cancer cells is in agreement with previous studies [33,34]. Meanwhile, a recent study revealed that the induction of the phosphorylation of IKKα by the ectopic overexpression of MALT1 facilitated the NF-κB subunits’ (p50 and p65) nuclear translocation to promote prostate cancer cell proliferation, invasion, and tumor growth in vitro and in vivo [30]. The immunoblot and NF-κB activity assays of this study demonstrated that the induction of androgen in the nuclear translocation of NF-κB signaling partly depends on the upregulation of MALT1 in LNCaP cells, indicating that the upregulation of NF-κB signaling by androgen induces PSA and NDRG1 gene expression via MALT1 in ARFL-positive prostate cancer cells (Figure 3). In agreement with previous studies, which explored how androgen dramatically increased the NDRG1 protein expression in LNCaP cells [35,40] and prostate cancer [41], the results of the present study confirmed further that R1881 stimulation significantly induces NDRG1 in LNCaP cells. Accordingly, our study suggested that a putative R1881/AR/MALT1/NF-κB-signaling pathway is involved in the regulation of PSA expression in ARFL-positive prostate cancer cells.

Androgen certainly has no effect on the MALT1 expression in ARFL-null PC-3 cells, although androgen upregulated the NDRG1 expression in the same cells, confirming that AR activity is indeed preserved in ARFL-overexpressed PC-3 cells. Unexpectedly, androgen did not influence the MALT1 expression in ectopic ARFL-overexpressed PC-3 cells (Figure 4A,B). Since studies have suggested a positive correlation between ARv7 expression and NF-κB activity in human benign prostatic hyperplasia and CRPC [21,37,42], we continued to study whether ARv7 was involved in the upregulation of MALT1-inducing NF-κB activity in androgen-independent prostate cells.

Unlike ARFL, ARv7 lacks a regulatory ligand binding domain and a unique encoded 16 amino acid sequence is inserted by the nucleotide, which is in the intron of the full-length AR transcript [43]. Studies have identified ARv7 as a tumor marker and as being associated with either prostate cancer aggressiveness or CRPC development [44,45,46]. The results of RT-qPCR assays (Figure 4C) indicated that only LNCaP and 22Rv1 cells express measurable ARFL and ARv7 mRNA instead of PZ-HPV-7, CA-HPV-10, PC-3, and DU145 cells; moreover, 22Rv1 cells express endogenously abundant ARv7. These results are in agreement with previous reports [43,47,48]. Both ectopic overexpressed-ARv7 22Rv1 and PC-3 cells induced NDRG1 expression compared to mock-transfected 22Rv1 and PC-3 cells, respectively; in addition, the ectopic overexpression of ARv7 induced PSA expression in 22Rv1 cells under androgen ablation conditions (Figure 5). Similar results are found in one study which showed that ectopic ARv7 overexpression in LNCaP cells upregulated the expression of NDRG1 and PSA in the absence of androgen [49]. Another study also explored an association between ARv7 and PSA RNA levels in the peripheral blood mononuclear cell fraction of prostate cancer patients [50].

The results of immunoblot assays with nuclear and cytoplasmic separation indicated that the ectopic overexpression of ARv7 upregulated MALT1 expression, which induced the phosphorylation of IκBα in cytosol and enhanced the translocation of NFκB subunits (p50 and p65) into the nucleus (Figure 5). This is the first study to verify that inducing NF-κB activity by ARv7 is through the upregulation of MALT1 in both 22Rv1 and PC-3 cells, even though the correlation between ARv7 expression and the NF-κB activity has been discussed [21,37,42]. MALT1 has been defined recently as an oncogene in human prostate cancer cells which enhances cell invasion and cell growth in vitro and in vivo [30,35]. The results of the in vitro cell proliferation and invasion assays showed that the ectopic overexpression of ARv7 enhanced cell proliferation and invasion in both 22Rv1 and PC-3 cells (Figure 6). These results are in agreement with previous studies, which showed the initiation of the growth of 22Rv1 cells by ARv7 [10,51]. Studies have found that high levels of ARv7 are correlated with prostate bone metastasis in vivo, and the epithelial-mesenchymal markers for cell invasion were identified as ARv7 target genes in both LNCaP and 22Rv1 cells in vitro [52,53,54]. However, none of the reports showed any molecular function of Arv7 in PC-3 prostate cancer cells. The results of our EdU proliferation and xenograft animal study (Figure 6 and Figure 7) confirmed further that ectopic ARv7 overexpression in ARv7-null PC-3 cells elevated cell growth in vitro and in vivo. Further study using a PC-ARv7 model to discover more molecular mechanisms of ARv7 in prostate bone metastasis is warranted, since the PC-3 cells have the characteristics to generate osteolytic bone lesions in a knee injection experiment [55].

Early studies have suggested that most prostate tumors and cell lines that express Arv7 also exhibit ARFL [43,49]. Other studies have implicated further that constitutively active ARv7 expression in CRPC required ARFL, and that the cell growth of the CRPC cell lines, LNCaP95 and 22Rv1, relied on both ARv7 and ARFL [10,51,56]. Thus, studies on ARv7 activity in CRPC have usually used the CRPC subcutaneous cell lines from LNCaP and 22Rv1 as the in vitro cell model. This study focused on the crosstalk among androgen, ARFL, ARv7, and NF-κB activities; therefore, we used not only LNCaP and 22Rv1 cell models to identify the androgen/ARFL/MALT1/NF-κB-signaling pathways but also 22Rv1 and PC-3 cell models to determine the ARv7/MALT1/NF-κB-signaling pathways. Our results indicated that the ectopic overexpression of ARv7 into 22Rv1 (ARv7-positive and ARFL-positive) and PC-3 (ARv7-negative and ARFL-negative) cells upregulated MALT1/NF-κB activities to enhance cell invasion and cell growth in vitro and in vivo. Since ARFL and ARv7 have demonstrated different ways of stimulating transcriptional activity in CRPC [11,45], further study using our cell model to explore new transcriptomes of ARv7 is warranted.

## 4. Materials and Methods

### 4.1. Cell Lines and Cell Culture

The non-metastatic cells, PZ-HPV-7 and CA-HPV-10, and the metastatic prostate cancer cells, LNCaP, 22Rv1, PC-3, and DU145, were from the Bioresource Collection and Research Center (BCRC, Hsinchu, Taiwan) and cultured as in a previous study [35]. The LNCaP cells are androgen-dependent and the 22Rv1 cells are androgen-independent cell lines; they are both androgen receptor full length (ARFL)-positive and androgen receptor splicing variant 7 (ARv7)-positive cells. The other cell lines contain neither ARFL nor ARv7. The PCJ cell line was subcultured from PC-3 prostate cancer cells, which were selected by the PSA reporter assay, as stated previously [57]. The cells were maintained in an RPMI 1640 medium (Life Technologies; Gaithersburg, MD, USA) with 10% fetal bovine serum (FBS; HyClone Laboratories, Inc., Logan, UT, USA). The charcoal-dextran-treated FBS (CD-FBS) was prepared as described previously [57]. Methyltrienolone (R1881), a synthetic androgen agonist, was from NEN Life Sciences (Boston, MA, USA). Enzalutamide (MDV3100), an AR antagonist, was from Sigma (St. Louis, MO, USA). For the studies of R1881 and/or MDV3100 treatments and the ectopic overexpression of ARv7, the cells were cultured in the phenol red free RPMI 1064 medium (Life Technologies; Gaithersburg, MD, USA) with 10% CD-FBS.

### 4.2. Immunoblot Assays

Equal amounts (20 μg or 40 μg) of the cell extracts were separated on a 10% or 12% dodecyl sulfate polyacrylamide gel electrophoresis (SDS-PAGE) gel. The blotting membranes were probed using an antiserum of ARv7 and ARFL (MA5-13426, Invitrogen, Carlsbad, CA, USA), MALT1 (EP603Y, Abcam, Cambridge, MA, USA), NF-κB p50 (06-886, Merck Millipore, Burlington, MA, USA), NF-κB p65 (06-418, Merck Millipore, Burlington, MA, USA), Lamin B1 (D9V6H, Cell signaling Technology Inc., Danvers, MA, USA), IκB-α (#9242, Cell signaling Technology Inc.), p-IκB-α (#2859, Cell signaling Technology Inc.), GAPDH (6C5, Santa Cruz Biotechnology, Dallas, TX, USA), NDRG1 (42-6200, Thermo Fisher Scientific Inc., Vilnius, Lithuania), PSA (A0562, Dako Denmark A/S, Glostrup, Denmark), or β-actin (T0022, Affinity bioscience, Cincinnati, OH, USA). The band intensities in the blot membrane were detected by the Western Lightning^TM^ Plus Chemiluminescence detection system (PerkinElmer Inc., Waltham, MD, USA), recorded by the LuminoGraph II chemiluminescent imaging system (Atto Corporation, Tokyo, Japan), and analyzed by the Image J software (version 1.52a).

### 4.3. Reverse Transcription-Polymerase Quantitative Chain Reaction (RT-qPCR)

The cDNAs were synthesized using the superscript III pre-amplification system (Invitrogen) after the total RNA was extracted from the cells using a TRIzol reagent (ambion, Life technologies, Carlsbad, CA, USA). The ARv7 primers (5′-GGAAATGTTATGAAGCAGGGATG-3′ and 5′-GGTCATTTGAGATGCTTGCA), ARFL primers (5′-TGCAGCCTATTGCGAGAGA-3′ and 5′-TGATCTCTGCCATCATTTCC), and 18S (5′-ACCGCAGCTAGGAATAATGGA-3′ and 5′-GCCTCAGTTCCGAAAACCA-3′) were used according to the previous report [50]. The qPCR reactions were performed in a 12 μL reaction volume that consisted of 6 μL of 2 x iQ^TM^ SYBR Green supermix (Bio-Rad Laboratories, Foster City, CA, USA), 1 μL of the cDNA sample, 1 μL of gene-specific primers (5 μM), and 4 μL of H_2_O. For the MALT1 and NDRG1 assays, TaqMan^TM^ gene expression master mix and FAM dye-labeled TaqMan MGB probes for human MALT1 (Hs01120052_m1), NDRG1 (Hs00608387_m1), and β-actin (Hs01060665_g1) from Thermo Fisher Scientific Inc. (Vilnius, Lithuania) were used as described previously [35]. The qPCR analysis was performed using a CFX Connect Real-PCR system (Bio-Rad Laboratories, Foster City, CA, USA). The cycle threshold (Ct) values for the target genes were normalized against the 18S or β-actin control probe to calculate the mean cycle threshold (ΔCt) values using the Bio-Rad CFX manager 3.1 (Bio-Rad Laboratories, Foster City, CA, USA).

### 4.4. EdU and Ki67 Flow Cytometry Assay

The cells were cultured in a serum-free medium for 24 h, then incubated for another 24 h in an RPMI-1640 medium with 10% FBS before incubation with EdU (5-ethynyl-2′-deoxyuridine; 10 μM) for 2 h. Subsequently, the cells were collected by trypsin-EDTA and centrifuged at 500× *g* for 10 min, then analyzed using Click-iT EdU flow cytometry assay kits (Thermo Fisher Scientific Inc., Waltham, MA, USA), as described previously [35]. For the Ki67 proliferation assay, the cells were permeabilized with 0.2% Triton X-100 for 10 min and blocked in 1% bovine serum albumin (A7906, Sigma-Aldrich Co., St. Louis, MO, USA) in a PBS solution. After an hour’s incubation, the cells were stained with a Ki-67 antibody conjugated PE (Ki67-PE) for 30 min in the dark. The proportion of EdU- or Ki-67-positive cells was measured using flow cytometry (Attune NxT acoustic focusing cytometer, Thermo Fisher Scientific Inc., Waltham, MA, USA).

### 4.5. Expression Vector Constructs and Stable Transfection

The expression vector containing the coding region of human ARv7 cDNA (OHu102656; GenScript, Piscataway, NJ, USA) was used. The human MALT1 cDNA (HG11618-UT) and control pCMV3 vector were purchased from Sino Biological Inc. (Beijing, China). The full-length AR (ARFL) expression vector and pcDNA3 vector were constructed as described previously [58]. The ARv7 expression vectors were introduced into PC-3 and 22Rv1 prostate cancer cells, respectively, by electroporation using the ECM 830 (BTX, Holliston, MA, USA) set on a single pulse setting for 70 msec at 180 V. The mock-transfection cells were transfected with control pcDNA3 expression vectors and clonally selected in the same manner as that of the gene-overexpression cells.

### 4.6. Gene Knockdown

The MALT1 shRNA lentiviral transduction particles (sc-35845-V, Santa Cruz Biotechnology, Santa Cruz, CA, USA) were transduced into LNCaP cells after the culture media was replaced with an RPMI-1640 medium plus 10% FBS and 5 μg/mL polybrene. The transduction cells were selected by incubating with 10 μg/mL puromycin dihydrochloride (Cyrus Bioscience, Seattle, WA, USA) for at least three generations, as described in the manufacture’s protocol. The mock-transfected cells were transduced with control shRNA lentiviral particles (sc-108080, Santa Cruz Biotechnology) and clonally selected in the same manner as that of the gene knockdown cells.

### 4.7. Reporter Vector Constructs, Transient Transfection, and Reporter Assay

The NF-κB reporter vector was from Clontech Laboratories, Inc., (Mountain View, CA, USA). The human MALT1 reporter vector contains the 5′-DNA fragment (−1 to −6313) of the human MALT1 gene, according to the sequence from GenBank (AP005018.1), and was synthesized and cloned into the pGL3-Basic reporter vector (pbGL3; Promega Biosciences, Madison, WI, USA) with Hind III sites, as previously described [35]. The reporter vectors, pPSABHE (−4801 to −3933 and −41 to −589), containing the 5′-flanking region of the human PSA promoter and the androgen-response element, were cloned as previously described [32]. The reporter vector and expression vectors were transiently transfected into 22Rv1 and PC-3 cells using the X-tremeGene HP DNA transfection reagent (Roche Diagnostics GmbH, Mannheim, Germany) and into LNCaP cells using the TransFast transfection reagent (Promega Biosciences). After transfection for 48 h, the reactants were washed twice in PBS and terminated by adding 200 μL of Luciferase Cell Culture Lysis Reagent (Promega Biosciences). The luciferase activity was determined in relative light units (RLU) using the synergy H1 microplate reader (BioTek Instruments, Inc., Beijing, China) and adjusted by the protein concentrations in the whole-cell extract, which was determined by a BCA protein assay kit (Pierce, Rockford, IL, USA).

### 4.8. Enzyme-Linked Immunosorbent Assay

The cells were incubated with 0.5 mL RPMI medium in a 24-well plate for 24 h. After incubation, the supernatants from each well were collected, and the PSA levels were analyzed using a PSA enzyme-linked immunosorbent assay kit (EL10005; Anogen, Mississauga, ON, Canada) as described by the manufacturer. The PSA levels in the conditioned media were adjusted by the protein concentrations in the whole-cell extract as described previously [35].

### 4.9. Matrigel Invasion Assay

The invasion ability of the PC-DNA, PC-ARv7, 22Rv1-DNA, and 22Rv1-ARv7 cells was determined through an in vitro Matrigel invasion assay as described previously [30].

### 4.10. NF-κB (p65) Transcription Factor Binding Assay

The cells were harvested with trypsin-EDTA. The cell pellets were washed twice with a phosphate buffer saline (PBS) and pelleted again by centrifugation at 500× *g* for 5 min. The nuclear and cytoplasmic fractions were separated by the NE-PER^TM^ nuclear and cytoplasmic extraction kit (Thermo Fisher Scientific Inc., Waltham, MA, USA). The NF-κB (p65)-binding activity was determined by the NF-κB (p65) Transcription Factor Assay kit (Cayman Chemical, Ann Arbor, MI, USA) as described previously [35]. Briefly, the nuclear extracts were incubated with a consensus dsDNA sequence at 4 °C overnight, and then the samples were incubated with a p65 primary antibody for another 1 h followed by a goat anti-rabbit HRP conjugate. After treatment with the Transcription Factor Developing Solution, the p65 binding activity was measured at an absorbance of 450 nm using the synergy H1 microplate reader (BioTek Instruments, Inc., Beijing, China).

### 4.11. Xenograft Animal Model

The xenograft animal studies were approved by the Chang Gung University Animal Research Committee (CGU106-157). The four-week-old male nude mice (BALB/cAnN-Foxn1) were from the animal center of the National Science and Technology Council (Taipei, Taiwan). The cells (PC3-DNA and PC3-ARv7) were detached from the cell flask using a Gibco Versene solution (Life Technologies, Grand Island, NY, USA) and washed with an RPMI 1640 medium with 10% FBS. The cell pellets were washed and re-suspended with PBS. The 6 × 10^6^ cell/100 μL cells of PC-DNA and PC-ARv7 were injected subcutaneously on the lateral back wall close to the shoulder of each mouse after the mice were anesthetized intra-peritoneally. The growth of the tumors was measured with a Vernier caliper every 3 days, and the tumor volume was determined by the formula, volume = length × width × width/2, as described previously [30]. According to the guidelines for laboratory animal facilities and care from the Council of Agriculture, Executive Yuan, Taiwan, the study of xenograft animals should be terminated when the diameter of tumors is up to 1.5–2.0 cm. The tumors of the xenograft animals were recorded and weighed after the mice were sacrificed. The collected tumors were lysed with a protein lysis buffer or Trizol reagent for further analysis of the mRNA or protein expression of the target genes.

### 4.12. Statistical Analysis

The statistical significance was determined through a *t*-test analysis and one-way ANOVA using the SigmaStat program for Windows version 2.03 (SPSS Inc., Chicago, IL, USA). Multiple comparisons were conducted using ANOVA with Tukey’s post hoc test. A significant difference was determined by *p*-value (*, *p* < 0.05, **, *p* < 0.01).

## 5. Conclusions

In this study, we demonstrated that androgen-induced MALT1 expression facilitates the nuclear translocation of the NF-κB subunits, p50 and p65, to stimulate the gene expression of PSA and NDRG1, indicating a novel androgen/ARFL/MALT1/NF-κB-signaling pathway presented in ARFL-positive but not ectopic ARFL-overexpressed AR-negative prostate cancer cells. ARv7 enhances the MALT1/NF-κB-signaling pathway in an androgen-independent way to motivate cell proliferation, invasion, and tumor growth in human androgen-independent prostate cancer cells in vitro and in vivo.

## Data Availability

Not applicable.
